# Impact of objectively-measured sleep duration on cardiometabolic health: A systematic review of recent evidence

**DOI:** 10.3389/fendo.2022.1064969

**Published:** 2022-12-19

**Authors:** Tadesse Asmamaw Dejenie, Markeshaw Tiruneh G/Medhin, Fitalew Tadele Admasu, Getachew Asmare Adella, Engidaw Fentahun Enyew, Zemene Demelash Kifle, Mohammed Abdu Seid, Misganaw Asmamaw Mengstie, Endeshaw Chekol Abebe

**Affiliations:** ^1^ Department of Biochemistry, School of Medicine, College of Medicine and Health Sciences, University of Gondar, Gondar, Ethiopia; ^2^ Department of Biochemistry, College of Health Sciences, Debre Tabor University, Debre Tabor, Ethiopia; ^3^ Department of Reproductive health and nutrition, School of public health, Woliata Sodo University, Woliata Sodo, Ethiopia; ^4^ Department of Human Anatomy, School of Medicine, College of Medicine and Health Sciences, University of Gondar, Gondar, Ethiopia; ^5^ Department of Pharmacology, School of Pharmacy, College of Medicine and Health Sciences, University of Gondar, Gondar, Ethiopia; ^6^ Department of Physiology, College of Health Sciences, Debre Tabor University, Debre Tabor, Ethiopia

**Keywords:** cardiometabolic health, type 2 diabetes, cardiovascular disease, objective, sleep duration

## Abstract

Cardiometabolic disease is a spectrum of diseases including, cardiovascular diseases, and metabolic syndrome. It is the leading cause of morbidity and mortality worldwide, with premature deaths being preventable. Currently, sleep has emerged as a potential target for cardiometabolic disease prevention. Several epidemiological studies have provided ample evidence that objectively measured short sleep duration increases the risk of cardiometabolic disease. However, the findings are inconsistent, and few studies measure sleep duration on cardiometabolic profiles objectively. Therefore, in this review, we focused on the recently published literature that explored the association between objectively measured sleep duration and cardiometabolic profiles (cardiovascular diseases, type 2 diabetes mellitus, and metabolic syndrome), seeking more insights regarding the applicability and, in turn, the impact of objectively measured sleep duration on cardiometabolic health, which is relatively understudied. We retrieved the information manually from PubMed, Google Scholar, HINARI, and the Cochrane Library from 2015 to 2022 using appropriate search terms, we included 49 articles. In this review, we found a strong relationship between objectively measured sleep duration and the risk of cardiometabolic disease, indicating that objectively measured short sleep durations increase cardiometabolic risks. In general, the association between objectively measured sleep duration and increased cardiometabolic risks (CMR) has been well-documented in higher-income countries. Several studies found that longer sleep duration was associated with a more favorable cardiometabolic profile in early adolescence, independent of other risk factors. On the other hand, objectively measured short sleep duration is associated with adverse cardiometabolic health outcomes such as coronary heart disease, hypertension, type 2 diabetes mellitus, and metabolic syndrome.

## 1 Introduction

Cardiometabolic disease is a spectrum of diseases including, cardiovascular diseases, and metabolic syndrome (1). Currently, it is the main contributor to morbidity and mortality globally with premature deaths being preventable. Factors contributing to cardiometabolic diseases are varied and influenced by environmental, social, political, and commercial determinants of health (2). The American College of Cardiology defines cardio-metabolic risks (CMR) as a collection of interconnected factors including hypertension, elevated fasting blood sugar, dyslipidemia, abdominal obesity, and elevated triglycerides (3). Based on the American Heart Association (AHA), in collaboration with the National Institutes of Health in 2019, the global prevalence of cardio-metabolic risks such as obesity (39.6%), hypertension (45.6%), diabetes mellitus (9.8%) and metabolic syndrome was found to be 21% (4). The aforementioned factors were thought to be major contributors to cardiovascular disease (CVD), which is responsible for 15% of global disability-adjusted life years (DALYs) and 30% of all deaths (5). The pooled prevalence of metabolic syndrome in Ethiopia is found to be 34.89%, with the weighted pooled prevalence of metabolic syndrome being higher in females (36.74%) compared to males (22.22%) (6). Subgroups at increased risk are type 2 diabetes patients, hypertensive patients, psychiatric patients, and HIV patients on HAART (7).

Sleep is an essential biological and behavioral process that plays a substantial role in maintaining good health and quality of life. It is controlled by diurnal, homeostatic, and neurohormonal mechanisms (8). Researchers have identified sleep as an essential modulator of cardiovascular function, blood glucose regulation, and hormonal secretion. Furthermore, sleep duration has an impact on metabolic hormones, body weight, the autonomic nervous system, the coagulation system, endothelial function, and metabolic regulation (9). Healthy sleep is characterized by many dimensions, including adequate duration, high quality, appropriate timing, and the absence of sleep disorders. Not getting enough sleep at night is generally associated with daytime sleepiness, daytime fatigue, depression, poor daytime functioning, and other health and safety problems (10).

In general, individuals’ sleep quality and quantity varies depending on age, sex, genetic factors, occupation, educational level, socioeconomic status, race, family relationships, and pathological conditions (11). Several studies also report that, in comparison with whites, African Americans are more likely to have sleep delays and short sleep durations. A delayed sleep phase was more likely to be observed among African American individuals with a morning or intermediate chronotype than among white individuals with the same chronotype (12, 13). Cespedes et al. report that objective sleep timing, including weekday time in bed, weekday time asleep, weekend time in bed, weekend time asleep, weekday mid-sleep time, weekday sleep duration, weekday in bed duration, weekend sleep duration, and weekend in bed duration, all differ significantly between African American and white participants (**Table 1**) (15). Based on the US National Sleep Foundation, adequate sleep recommendations also vary across lifespans and are inversely related to age, with 7–9 hrs recommended for adults (26, 27). However, in recent decades, the average sleep duration has decreased across the globe, and this has negatively impacted cardiometabolic health (11).

**Table 1:** summary of studies on objective sleep duration and cardiometabolic health adverse outcomes.

Type of Study	Study participants	Sleep measurement	Study settings	Study outcomes	Reference
Demographic Surveillance study	167 adults (≥ 40 years)	Using Actigraphy sleep measurement	South African	Short and fragmented sleep was significantly associated with poor cardiometabolic health outcomes.	Cook and Mohlabe, 2022 (14)
Cross-sectional study	829 sample of children entering adolescence, period	Using Actigraphy sleep measurement	*Massachusetts*	Longer sleep duration in early adolescence was associated with more favorable cardiometabolic health.	Cespedes et al., 2018 (15)
A pilot study in a rural African setting	139 adults (≥40 years)	Using actigraphy sleep measurement	South Africa	The objectively measured short sleep duration was significantly associated with HOMA-IR and showed a linear relationship.	Cook et al, 2021 (16)
					
Cohort study	384 Mexican adolescents from a birth cohort study	Using actigraphy sleep measurement	Mexico City	Both the sleep duration and the timing were independently associated with insulin resistance, with the association being stronger in females. Shorter sleep duration was associated with higher log HOMA-IR	Chen et al., 2021 (17)
Cohort Study	Overall, 1,852 participated in the study.	Using actigraphy sleep measurement	Northeastern United States	Sleep health was associated with lower cardiometabolic disease risks.	Buxton et al., 2018 (18)
Cross-sectional Study	255 adults	Polysomnography sleep measurement	Chicago	After controlling for potential confounders, the odds of reporting hypertension increased more than threefold in objectively measured short sleep duration but was not significant in subjectively measured short sleep duration	Bathgate et al., 2016 (19)
Prospective study	145 adults	Polysomnography sleep measurement	USA	Bowman et al. provide evidence that subjective and objective measures of sleep differ in their ability to prospectively predict MetS.	Bowman et al., 2019 (20)
Longitudinal Study of Australian Children	2451 (Adults (n=1378), and Children, n= (1073))	polysomnography in both adults and children	Australia	Short sleep duration is strongly associated with higher BMI and MetS in children and higher SBP in adults.	Matricciani et al., 2020 (21)
prospective cohort study	245 indigenous Australians aged > 18 yrs	Actigraphy sleep measurement	Australia	According to Yiallourou et al., short sleep duration negatively impacts blood pressure, hemoglobin A1c, and cholesterol levels.	Yiallourou et al.,2021 (22)
Longitudinal Study	2380 (1043 children and 1337 adults)	Using actigraphy sleep Measurement	Australia	Actigraphy-based sleep profiles are associated with cardiometabolic profiles and good sleep patterns are associated with more favorable cardiometabolic health.	Matricciani et al., 2021 (21)
Cross-sectional study	1,268 individuals enrolled in this study	Using actigraphy sleep Measurement	Brazil	In adolescence, there was no association between sleep duration and cardiometabolic risk factors. However, they concluded that the associations may arise in other life phases as well.	Confortin et al., 2022 (23)
Community‐based, prospective cohort study	A total of 3810 participants were enrolled	Using polysomnography	China	Short sleep duration as objectively measured by polysomnography, was associated with an increased risk of cardiovascular disease.	Yan et al., 2021 (24)
Cohort study	A total of 1654 adults (aged 20–74 years) participated in this study	Using polysomnography total sleep time <6 hours	Penn State	Objectively measured short sleep duration predicts the prognosis of all-cause mortality in middle-aged adults with cardiometabolic risks.	Fernandez‐Mendoza et al., 2021 (25)

To assess sleep, a variety of tools are used. There are valid questionnaires and surveys that provide information on subjective sleep parameters (28), including quantity, latency to sleep onset, duration, and level of daytime sleepiness, such as the Pittsburgh Sleep Quality Index (29) and Epworth Sleepiness Scales (30). Even though self-reported sleep parameters are relatively easy to administer and inexpensive, methodological concerns and finding inconsistencies arise (31). Currently, objective sleep measuring tools are widely used mainly in developed countries. Among the objective sleep measuring tools, polysomnography (PSG) is a sleep laboratory test that provides detailed information on nocturnal physiology, including a recording of objective sleep architecture as well as measures of cardiopulmonary function (32). Actigraphy, which is another objective sleep measurement, evaluates sleep versus wake time with a small wrist-worn monitor and can more conveniently capture information (33). Importantly, there may be major differences in outcomes when comparing subjective versus objectively recorded sleep quantity and quality (27). Currently, several studies have reported that individuals with sleep disorders and objectively measured short sleep duration were found to have a higher risk of adverse cardiometabolic profiles in objectively recorded sleep, although no significant association was observed using subjectively assessed sleep (25, 34).

In this review, we focused on the recently published literature that explored the association between objectively measured sleep duration and cardiometabolic diseases (cardiovascular diseases, type 2 diabetes mellitus, and metabolic syndrome), seeking more insights regarding the applicability and, in turn, the impact of objectively measured sleep duration on cardiometabolic health, which is relatively understudied.

### 1.1 Methods

**Figure 1:**
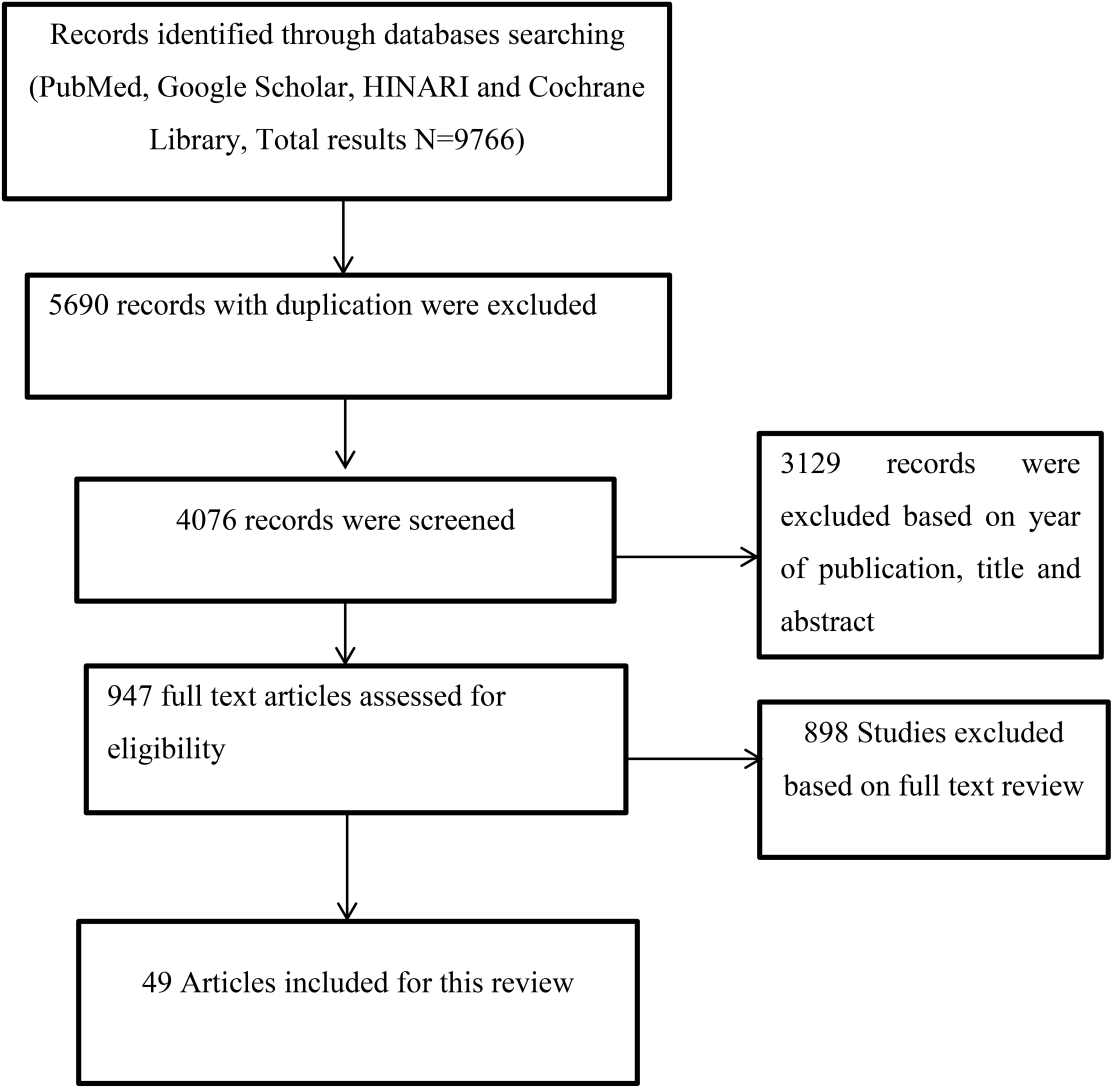
Flowchart of the literature search.

The information was manually retrieved from PubMed, Google Scholar, HINARI, and the Cochrane Library from 2015 to 2022 (**Figure 1**), to assess studies that examined the relationship between objectively measured sleep duration and cardiometabolic disease (metabolic syndrome, cardiovascular disease, and type 2 diabetes mellitus). We used appropriate search terms from 2015 scoping searches, target references, and browsing of database thesauruses (objective sleep duration and cardiometabolic diseases) A basic search strategy was developed for PubMed and modified accordingly for other research engines using the following search criteria; objective sleep duration OR cardiometabolic adverse outcomes OR cardiovascular disease OR metabolic syndrome OR type 2 diabetes mellitus; OR objective sleep duration AND cardiometabolic adverse outcomes AND cardiovascular disease AND metabolic syndrome AND type 2 diabetes mellitus. Those articles written in English and which were based on objectively measured sleep duration and were recently published from 2015 to 2022 were included for this review. On the other hand, articles written in languages other than English, based on self or parental sleep assessments, and published prior to 2015, with the exception of the definition of terms and proposed mechanisms that show the link between objectively short sleep duration and cardiometabolic health adverse outcomes, which can be the same for both objectively and subjectively measured sleep duration, were excluded from the review.

## 2 Main text

### 2.1 Sleep duration and cardiometabolic adverse outcomes

A strong relationship between sleep duration and the risk of cardiometabolic disease has been found in several studies, indicating that objectively measured short sleep durations increase cardiometabolic risks (35, 36). In the general, the association between objectively measured sleep insufficiency and increased cardiometabolic risks (CMR) has been well-documented in higher-income countries (37). However, it is relatively limited in low-income countries such as Sub-Saharan Africa (9, 38). Thus, little is known about the prevalence and consequences of objectively measured sleep disorders in most African populations, and their association with CMR has not yet been well documented (37). In fact, more research has been conducted on the relationship between sleep and cardiometabolic health in developed nations. For instance, in a cross-sectional study of 829 adolescents using wrist actigraphy recording, Feliciano et al. found that Independent of other risk factors, optimum sleep duration and better sleep quality were linked to a more favorable cardiometabolic profile in early adolescence (15). However, some studies also showed that very long sleep duration was associated with poor cardiometabolic health outcomes, stating that there were increased odds of diabetes among long sleepers (>8 hr) and that it was also associated with a higher risk of CVD mortality (24). Evidence studies suggest a U-shaped association between sleep duration and T2DM and show that long sleepers have almost 60% higher odds of developing T2DM compared to normal sleepers (39). On the other hand, objectively measured short sleep duration is associated with adverse cardiometabolic health outcomes such as coronary heart disease, hypertension, type 2 diabetes mellitus, and metabolic syndrome (40). In the following sections, the authors have discussed each component of cardiometabolic disease and its association with objectively measured sleep duration.

#### 2.1.1 Cardiovascular disease

The leading cause of death in the world today is cardiovascular disease (CVD), including coronary heart disease (CHD), stroke, peripheral arterial disease, deep vein thrombosis, and pulmonary embolism (41, 42). By addressing modifiable risk factors, such as reducing excessive dietary salt, improving a poor diet, lowering obesity, avoiding excessive alcohol consumption, increasing physical activity, and improving socioeconomic conditions, premature deaths from cardiovascular disease can be prevented (43). This is the most cost-effective intervention for reducing cardiovascular disease-related morbidity and mortality and improving cardiovascular health.

According to the Coronary Artery Risk Development in Young Adults (CARDIA) cohort study conducted in Chicago, there is evidence of an association between objectively measured sleep duration and cardiometabolic disease (43). From this report, 18- to 30-year-old individuals were participated and were followed up for five years. The sleep metrics were measured using wrist actigraphy, and to measure the calcifications of their coronary arteries, a CT scan was used. After excluding the possible confounding factors, participants with a sleep habit of 5 hours or less had a sharp increase in their risk of developing coronary calcifications, which is in turn a risk factor for myocardial infarction. Women are more affected than men, although the possible justification of gender differences is not well stated. Similar to what was reported, individuals who regularly slept less than 5 or 6 hours were more likely to experience episodes of coronary heart disease and develop stroke in the long run than people who typically slept for about 6 to 8 hours each night (44). If the short sleep is accompanied by poor sleep quality, the risk is greater. In general, few studies have investigated the associations of objectively measured sleep duration with incident cardiovascular diseases (CVDs). A prospective cohort study of 12,770 people in the United Kingdom discovered that getting enough sleep is associated with a lower risk of cardiovascular disease in people with diabetes (45). A cross-sectional study involving a sample of 893 adults found that shorter sleep duration, as determined by wrist actigraphy, is associated with higher SBP and possibly cardiovascular risk. Objectively measured short sleep duration may therefore be a modifiable behavioral target for reducing cardiovascular risk (46). Yadav et al. also showed that objectively measured short sleep duration was consistently associated with the occurrence of hypertension in middle-aged participants (47). The findings were in line with previous studies carried out by Bertisch et al. which also reported that short sleep duration was associated with hypertension compared to individuals who had normal sleep duration (48). Another prospective study of 1715 Korean adults aged 40 to 70 years found that objectively short sleep duration is independently associated with the development of hypertension among middle-aged and elderly Korean adults (47). Thus, the study encourages clinicians and researchers to promote sound sleep practices in society by giving evidence that explains the association between objective short sleep duration and hypertension as a risk predictor of the incidence of cardiovascular disease.

Hypertension is a known risk factor for cardiovascular disease and leads to disability and death. It is often described as a “silent killer” because it rarely causes symptoms (49). Currently, studies prove a significant association between short sleep (less than 5 hours per night) and an increased risk of hypertension (19, 50). In the Penn State Cohort study, Bertisch et al. showed that objectively measured short sleep duration is a clinically significant risk factor for the development of hypertension independent of other risk factors such as age, race, obesity, diabetes mellitus, smoking, caffeine, or alcohol consumption (48). Using a cross-sectional, observational study design of 255 adult volunteers, Bathgate et al. reported that objectively measured short sleep duration of < 6 hr was associated with an increased risk of hypertension development as compared to individuals with a sleep duration of >6 hrs (51). However, no significant risk was observed using subjectively measured sleep duration groups in this study. Finally, this study concluded that objectively short sleep duration increased the likelihood of hypertension more than three times, taking into account possible confounding variables, and, in turn, increased the risk of cardiovascular disease (51).

#### 2.1.2 Metabolic syndrome

The term “metabolic syndrome” refers to a group of metabolic risk factors including glucose intolerance, dyslipidemia, hypertension, and central obesity that are associated with an increased risk of type 2 diabetes mellitus (T2DM) and cardiovascular disease (CVD), including glucose intolerance, dyslipidemia, hypertension, and central obesity (52). According to the Joint Interim Statement (JIS) harmonized, the following three components are required for the definition of Metabolic Syndrome (MetS), of which waist circumference is not a prerequisite: High resting blood pressure (≥ 130/85 mmHg) or on hypertension treatment; ≥ 5.6 mmol/l fasting blood sugar or on diabetes treatment: Elevated waist circumference (WC): Females ≥92 cm, males ≥86 cm; elevated triglycerides (TG): ≥1.7 mmol/l and reduced high-density lipoprotein cholesterol (HDL-C): men < 1.0 mmol/l, women < 1.3 mmol/l (53). Most previous studies exploring the relationship between sleep duration and MetS-related morbidity and mortality were inconsistent and underwhelming (54–56). For instance in In a cross-sectional study of Chinese children and adolescents, Duan et al. reported that objective short sleep duration is associated with higher odds of metabolic syndrome and abdominal obesity. However, the relationship between short sleep duration and MetS in children and adolescents has been inconsistent (56). In a population-based study, Fernandez-Mendoza et al. demonstrated the PSG and reported that objectively measured short sleep duration modifies the elevated risk of CVD mortality associated with MetS. Importantly, this is primarily caused by the high blood pressure and glucose dysregulation components of MetS, which suggests that objectively short sleep duration in those with MetS may be related to the degree of central autonomic and metabolic dysfunction (57). On the other hand, Cook et al. found a significant non-linear (U-shaped) association between short sleep duration and JIS-MetS risk (p = 0.0308) (**Table 1**) (16). The findings of this study are consistent with the study conducted by Rae et al. (9). According to the authors, this was the first actigraphy-measured sleep and cardiometabolic health study from a rural South African setting. Future clinical trials should investigate whether long-duration sleep improves the prognosis of people with MetS.

The global prevalence of obesity has increased dramatically over the past few decades, and the World Health Organization has declared it a global epidemic (58). The rise in obesity is paralleled with a decrease in sleep duration. Most of the previous studies were predominantly cross-sectional; that is, they measured both sleep duration and the presence of obesity at the same time. Therefore, a possible relationship cannot support a causal association. More recently, however, objective measures of sleep using actigraphy and more specific measures of adiposity (fat mass versus lean body mass) appear to support the link between sleep deprivation and obesity (59). Experimental studies provide a good explanation of the plausible mechanism of how short sleep leads to obesity. Chronic short-term sleep results in increased energy intake and decreased energy expenditure by activating hormonal responses that regulate appetite and energy balance (60). During sleep deprivation, there are reciprocal changes in leptin, a hormone that regulates energy storage and satiety, and ghrelin, a hormone that increases appetite (**Figure 1**) (61). These two hormones regulate the hunger and satiety of individuals.

Several epidemiologic and longitudinal studies have reported that short sleep duration is a risk factor for the incidence of obesity. For example, in sex-and age-stratified analyses, Sluggett et al. indicated that short sleep duration increased the likelihood of being overweight or obese in children and adolescents compared to longer sleepers (62). In contrast to this study, a cohort sleep laboratory study using polysomnography (PSG) showed that objectively measured short sleep duration is not significantly associated with obesity (63).

#### 2.1.3 Type 2 diabetes mellitus

Type-2 diabetes is the most common type of diabetes in the world, and it is characterized by the body’s inability to use glucose from circulation, resulting in hyperglycemia. This is caused by peripheral tissue resistance to the action of insulin to take up glucose into the cell (insulin resistance) or inadequate pancreatic insulin production in response to a glucose load (64).

Currently, several studies have revealed that sleep duration is associated with poor glycemic control (17, 65, 66). In a sample of 384 Mexican adolescents from a birth cohort study, Chen et al. reported that objectively shorter sleep duration and later sleep midpoint was associated with higher insulin resistance (HOMA-IR). In linear regression analysis, they found that shorter sleep duration was associated with higher log HOMA-IR after adjusting for age, sex, and sleep midpoint. In the sex-stratified models, the associations between insulin resistance and short sleep duration were more evident among girls (17). Another prospective cohort study of nulliparous women recruited between 16 and 21 weeks gestation found that shorter sleep duration and a later sleep midpoint using the actigraphy sleep measuring tool were associated with an increased risk of gestational diabetes. This study concluded that short sleep duration is the sole contributing factor to gestational diabetes in nulliparous women (67). This research was consistent with a cross-sectional study done in the UK with 2848 adult participants, which showed a link between short sleep duration and the incidence of diabetes mellitus (64). According to a population-based study conducted in 2021 on a Taiwanese population, short sleep duration increased the risk of T2DM. This study found that women who sleep for shorter periods have a higher risk of developing type 2 diabetes, which has an impact on the Homeostatic Model Assessment for Insulin Resistance (HOMA-IR) index (68). In this review, the authors observed that the association between sleep durations and increased CMR has been well-documented in higher-income countries (37). However, objective sleep measures are relatively limited in low-income countries such as Sub-Saharan Africa. The majority of sleep studies conducted in this region most frequently used self-reported sleep. Thus, little is known about the prevalence and consequences of sleep disorders in African populations, and their association with type 2 diabetes mellitus has not yet been well investigated, based on objective sleep measures, in most parts of African countries (37).

## 3 Discussion

In general, the association between objectively measured sleep duration using actigraphy or/and Polysomnography and increased cardiometabolic risks (CMR) has been well-documented in higher-income countries. However, studies in developing nation mainly parent- or self-reported sleep assessment tools, and very few studies have examined the association between objectively measured sleep duration and cardiometabolic health outcomes. For example, in a demographic surveillance study done in South Africa using actigraphy sleep measures, it was found that insufficient and fragmented sleep were significantly associated with poor cardiometabolic health outcomes (14) (**Table 1**). Another pilot study in a rural setting in South Africa using the same sleep measurements found that objectively measured short sleep duration was significantly associated with HOMA-IR and showed a linear relationship with type-2 diabetes mellitus (16).

A vast majority of studies conducted in developed nations used objective sleep assessment tools. For instance, a cross-sectional study using actigraphy sleep measurement in Massachusetts reported that longer sleep duration in early adolescence was associated with more favorable cardiometabolic health outcomes (15). Studies also showed that very long sleep duration was associated with poor cardiometabolic health outcomes (24), showing that there is a U-shaped association between sleep duration and cardiometabolic health outcomes (39). A cohort study conducted on Mexican adolescents found that both the objectively measured sleep duration and timing were independently associated with cardiometabolic risks, with the association being stronger in females (17) (**Table 1**). In line with this, a study conducted in the Northeastern United States found that healthy sleep was associated with lower cardiometabolic disease risks (18), which agreed with a study conducted in Chicago that indicated the odds of reporting hypertension increased more than threefold in objectively measured short sleep duration but were not significant in subjectively measured short sleep duration (19). Another prospective study in the United States using the same sleep measurement tool provided evidence that subjective and objective measures of sleep differ in their ability to prospectively predict cardiometabolic risk (20). On the other hand, a longitudinal study in Australia reported that objectively measured short sleep duration is strongly associated with higher BMI and MetS in children and higher SBP in adults (21). In the same study setting, another study on indigenous Australians aged > 18 years found that short sleep duration negatively impacts blood pressure, hemoglobin A1C, and cholesterol levels (22). On the one hand, a cross-sectional study that was conducted in Brazil concluded that there was no association between sleep duration and cardiometabolic risk factors and justified the notion that the associations may arise in other life phases as well (21). Besides, a study conducted at Penn State reported that objectively measured short sleep duration predicts the prognosis of all-cause mortality in middle-aged adults with cardiometabolic risks (25).

## 4 Conclusion and future perspectives

In this review, we noted that the impact of short sleep duration on cardiometabolic health is widely acknowledged and has been thoroughly examined in the literature over the past decades. There is a strong and consistent association between the duration of sleep, especially short sleep duration, and cardiometabolic health outcomes. A U-shaped relationship between sleep duration and the risk of cardiometabolic disease has been found in several studies, indicating that objectively measured very long and short sleep durations increase cardiometabolic risks.

We suggest that future studies should investigate population-based longitudinal associations between sleep and cardiometabolic health with the use of objective sleep measurements conducted for several days and at multiple time points over time. Meanwhile, based on the available evidence, we recommend that children and adolescents should get adequate amounts of good sleep in a regular pattern.

Furthermore, we suggest conducting more studies examining the associations between gender-stratified sleep patterns and cardiometabolic health as well as more studies to be performed in developing countries because most sleep research is conducted in developed nations, but cardiometabolic risk factors are more prevalent in developing countries. Besides the authors observed that many of the sleep studies on cardiometabolic health were conducted in older adults, but sleep-disorder-related factors, including duration, quality, and timing, may be emerging as early as childhood. As a result, further research should be performed on the role of childhood sleep insufficiency across the life course as a determinant factor for developing the adult cardiometabolic disease.

## Author contributions

All authors made a significant contribution to the work reported, whether in drafting or critical revision for important intellectual content; the conception, study design, execution, acquisition, and interpretation; or in all these areas; they took part in critically reviewing the article; gave final approval of the version to be published; agreed on the journal to which the article has been submitted; and agreed to be accountable for all aspects of the work. All authors contributed to the article and approved the submitted version.
